# Coupling Liquid Electrochemical TEM and Mass‐Spectrometry to Investigate Electrochemical Reactions Occurring in a Na‐Ion Battery Anode

**DOI:** 10.1002/smtd.202400365

**Published:** 2024-08-29

**Authors:** Kevyn Gallegos‐Moncayo, Nicolas Folastre, Milan Toledo, Hélène Tonnoir, François Rabuel, Grégory Gachot, Da Huo, Arnaud Demortière

**Affiliations:** ^1^ Laboratoire de Réactivité et Chimie des Solides (LRCS) CNRS UMR 7314 UPJV Hub de l'Energie 15 rue Baudelocque Amiens Cedex 80039 France; ^2^ Réseau sur le Stockage Electrochimique de l'Energie (RS2E) CNRS FR 3459 Hub de l'Energie 15 Rue Baudelocque Amiens Cedex 80039 France; ^3^ ALISTORE‐European Research Institute CNRS FR 3104 Hub de l'Energie Rue Baudelocque Amiens Cedex 80039 France

**Keywords:** foam, free‐anode, GC/MS, HC, In situ ec‐TEM (µ‐battery), NIB, NP30, plating, SEI

## Abstract

A novel approach for investigating the formation of solid electrolyte interphase (SEI) in Na‐ion batteries (NIB) through the coupling of in situ liquid electrochemical transmission electron microscopy (ec‐TEM) and gas‐chromatography mass‐spectrometry (GC/MS) is proposed. To optimize this coupling, experiments are conducted on the sodiation of hard carbon materials (HC) using two setups: in situ ec‐TEM holder and ex situ setup. Electrolyte (NP30) is intentionally degraded using cyclic voltammetry (CV), and the recovered liquid product is analyzed using GC/MS. Solid product (µ‐chip) is analyzed using TEM techniques in a post‐mortem analysis. The ex situ experiments served as a reference to for insertion of Na+ ions in the HC, SEI size (389 nm), SEI composition (P, Na, F, and O), and Na plating. The in situ TEM analysis reveals a cyclability limitation, this issue appears to be caused by the plating of Na in the form of a “foam” structure, resulting from the gas release during the reaction of Na with DMC/EC electrolyte. The foam structure, subsequently transformes into a second SEI, is electrochemically inactive and reduces the cyclability of the battery. Overall, the results demonstrate the powerful synergy achieved by coupling in situ ec‐TEM and GC/MS techniques.

## Graphical abstract

In this study, a novel approach for investigating the formation of solid electrolyte interphase (SEI) in Na‐ion batteries through the coupling of in situ liquid electrochemical TEM and gas‐chromatography mass‐spectrometry is proposed. To optimize this coupling, experiments are conducted on the sodiation of hard carbon materials using two different setups: in situ ec‐TEM holder and ex situ setup.

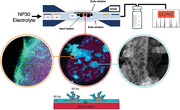

## 1 Introduction

The growing demand for energy storage devices has become a key issue for our well‐being. Among the solutions, Li‐ion batteries (LIBs) are widely used in portable consumer electronics and electric vehicles due to their high‐density energy, high cycle durability, and fast speed of charge. However, the increasing demand for LIBs has led to price's rise and increased pressure on Lithium.^[^ ^1^ ^]^ To find a cleaner and more sustainable alternative, interest has again been focused on NIBs. NIB technology was already explored in the 80s, but it was abandoned due to the better performance of LIBs.^[^ ^2^, ^3^, ^4^ ^]^

NIB is now considered a promising technology because Na is naturally abundant, cheap, and environmentally friendly. NIBs allow the use of Al foil as a current collector in both, anode and cathode, which, in comparison with LIB (use of Cu current collector at low voltages) represents a reduction in costs of fabrication.^[^ ^5^ ^]^ In addition, NIBs offer high power output and fast charging for applications such as electric bicycles, scooters, mopeds, robots, industrial tools, etc. NIBs also can be considered as an alternative to LIBs in terms of long‐term energy storage.^[^ ^6^ ^]^ Nevertheless, they are not competitive in terms of electrical energy density.^[^ ^7^ ^]^

Thus, in recent years, to improve their energy performance, different methods have been used to characterize NIBs during the charge/discharge process, such as XRD, XPS, NMR, SEM, and, TEM.^[^ ^2^, ^8^, ^9^ ^]^ However, the dynamic conditions within a battery had not been explored due to the requirement of a sealed working environment, until the advent of the in situ liquid ec‐TEM characterization technique.^[^ ^10^, ^11^, ^12^ ^]^ This is a powerful tool for real‐time liquid TEM imaging applications that provides a deeper understanding of the material's behavior in the electrochemical liquid environment, such as oxidation‐reduction reactions, nucleation, crystalline phase transformations, the kinetics of chemical species formation, and movement of nanometric objects in the fluid.^[^ ^2^, ^13^, ^14^ ^]^

The dynamic study of electrochemical regimes in NIBs with high spatial resolution at the nanoscale is fundamental to further understanding these energy storage systems. For example, Lutz L. et al. have evidenced, thanks to the in situ liquid ec‐TEM technique, the development of parasitic reactions in Na‐O_2_ batteries, which could be responsible for the low charge capacity and poor cycling stability of the batteries.^[^ ^12^ ^]^ On the other hand, to reinforce the in situ liquid ec‐TEM technique, we propose coupling it with mass‐spectrometry to analyze the products formed during the charge and discharge of NIBs. Studies carried out with GC/MS by G. Gachot et al. detected and accurately identified a wide range of volatile molecules resulting from battery degradation, demonstrating that CH_3_OLi is the initiator of electrolyte degradation in lithium‐ion batteries.^[^ ^15^, ^16^, ^17^ ^]^

By using the in situ liquid ec‐TEM and the GC/MS coupling, we intend to optimize the coupling and investigate the electrochemical behavior of the HC electrode in contact with the NP30 electrolyte.^[^ ^18^ ^]^ Thus, to improve the performance of the NIB, it is necessary to find optimal compromises between electrolyte compositions and electrode surface states, since the formation of the protective layer of SEI depends on them. The selection of appropriate electrolytes is particularly crucial as it governs the composition of the SEI, influencing its properties including ionic conductivity, electronic resistance, stability, and thickness.^[^ ^19^, ^20^ ^]^

Even though the electrolyte formulation is studied as a crucial factor for the quality of the life cycle, the SEI is not well known for NIBs. Previous studies^[^ ^21^ ^]^ indicate that secondary reactions, such as the degradation of electrolytes, take place in the first cycle of the battery, which leads to the formation of SEI on the hard carbon surface. From a thermodynamic point of view, the anodic stability of an electrolyte depends on its level of lowest unoccupied molecular orbital (LUMO). The electrolyte component with the lowest LUMO level will reduce first in contact with the HC electrode, possibly contributing to SEI formation.^[^ ^21^ ^]^

Several authors have studied the degradation and formation mechanisms of SEI and electrolyte degradation in LIB systems by using in situ liquid ec‐TEM and GC/MS analysis separately. For instance, Robert L. Sacci et al.^[^ ^22^ ^]^ studied the SEI formation using in situ liquid ec‐TEM, however, there was no reported analysis on the degradation of the electrolyte. In addition, other authors were interested in the comprehension of electrolyte degradation and used both, TEM and GC/MS techniques, but exclusively ex situ in coin cell batteries and without synergy between TEM and GC/MS.^[^ ^23^, ^24^, ^25^ ^]^ This study presents a novel investigation of SEI formation and electrolyte degradation during Na‐ion insertion into hard carbon materials in a NIB system. To our knowledge, this is the first report using a simultaneous approach involving ec‐TEM and GC/MS techniques. The synergistic combination of these techniques offers a promising methodology for gaining deeper insights into the underlying phenomena of NIBs. Furthermore, this approach can be extended to optimize other energy storage materials, thus expanding its potential as a valuable tool in the field of energy storage research.

## 2 Experimental Section

**Figure 1:**
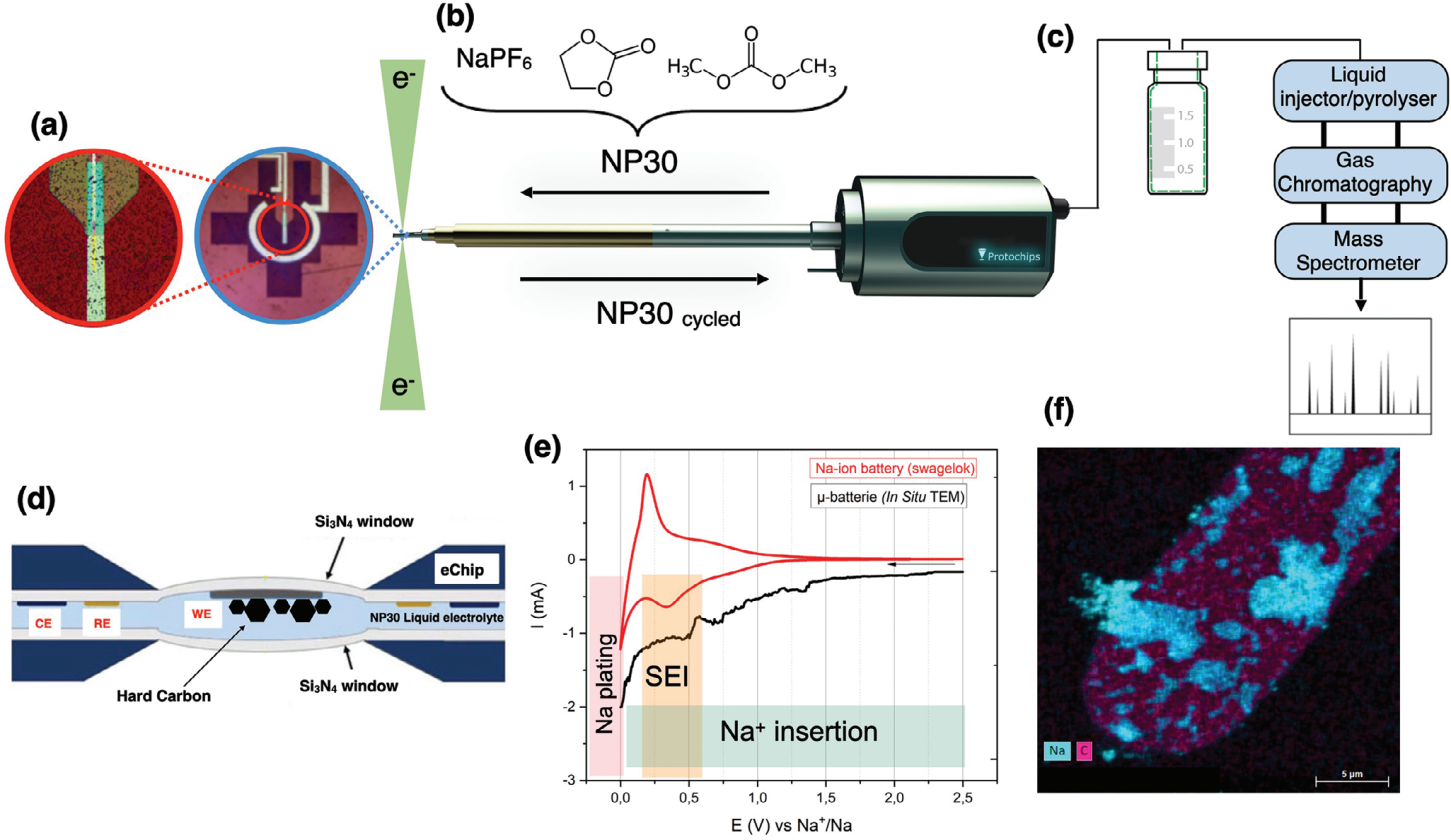
Methodology process for liquid electrolyte degradation analysis. a) real image of electrodes in a microchip for electrochemical measures, working electrode (WE), reference electrode (RE), and counter electrode (CE). b) Protochips sample holder for liquid ec‐TEM image in situ analysis. c) Hermetic flask for the cycled liquid electrolyte for further analysis and expected chromatograph after analysis of volatile liquid electrolyte in GC/MS. d) Schematic representation of in situ electrochemical liquid cell with Hard Carbon forming µ‐battery. e) Comparison of CV of Na‐ion battery versus µ‐battery, including Na^+^ insertion, SEI formation, and Na plating. f) Results from EDX‐STEM imaging in liquid TEM cell using µ‐battery.

For a better understanding of electrolyte degradation, an adapted configuration was conceived to perform close‐to‐reality battery cycling, as shown in **Figure** **1**. The liquid electrolyte NP30 (hexafluorophosphate NaPF_6_ 1 m, in a mix of ethylene carbonate and dimethyl carbonate EC:DMC 1:1 in mass ratio) was injected via micro fluid tubes to the ec‐TEM cell for cycling in an HC/Na half cell, as shown in Figure 1b,d. The electrochemical measurements were performed with a 3‐electrode system displayed in Figure 1a, and the results are presented in Figure 1e as a CV scanning between 0.0 and 2.5 V (vs Na^+^/Na), with a sweeping rate of 1 mV s^−1^. The OCV of the system is 2.49 V (vs Na^+^/Na) and the first reaction that takes place is reduction, then oxidation. Immediately after cycling, and while the cell is still inside the TEM chamber, TEM imaging was performed, and the cycled electrolyte was recovered after the first discharge in a hermetic flask for further analysis in GC/MS, as shown in Figure 1c. This process reduces the perturbation in each step of data acquisition, especially the further degradation of the electrolyte due to environmental exposure. It reflects as well a less biased result and, in consequence, allows a better interpretation of the phenomenon observed.

### 2.1 Half Coin Cell Battery

CV was performed in a half coin cell (HC/Na metal) to obtain a global view of the electrochemical behavior of the configuration anode/electrolyte used here as an experimental reference for the in situ investigation. The counter‐electrode was metallic Na. The electrolyte used for the coin cell was NP30 (NaPF_6_ 1 M, EC: DMC 1: 1 mass). Electrochemical analysis was performed using SP200 potentiostat. EC‐lab software was used for controlling the device. The scan conditions are those presented before, and the conditions are identical for all the electrochemical curves performed during this study.

### 2.2 EC‐TEM Electrode Preparation

The active material (commercial HC suspended in methanol) was deposited on the top e‐chip through the drop‐casting method (20–200 µl taken with a micropipette deposited over the µ‐chip and left to dry for 24 h at room temperature).

### 2.3 µ‐Battery Assembly

The head of the in situ liquid ec‐TEM sample holder contains a µ‐fluidic tubing system and an electrical wiring system for electrochemistry, as shown in Figure 1d. The electrochemical liquid cell, also called µ‐battery in this work, was prepared from the assembly of two seals: a bottom e‐chip and a top e‐chip, which are in contact with O‐ring polymer to guarantee a good sealing. There are 3 coplanar electrodes on the top e‐chip: a Working Electrode (WE) (glassy carbon), Reference Electrode (RE) (Pt), and Counter Electrode (CE) (Pt). The HC active material was placed on the glassy carbon surface, the WE of the µ‐chip. This assembly is held in place by a metal hood that is adjusted with a torque screwdriver.

### 2.4 TEM Analysis

TEM and STEM analyses were performed using TECNAI F20‐S‐TWIN, FEI device, and the parameters were adjusted as follows: 200 KV for the acceleration tension, OneView camera (Gatan), spot size 5, and a 70 µm condenser aperture. Image techniques applied over the sample were TEM image, EDX‐STEM (Scanning Transmission Electron Microscope Energy Dispersion X‐ray), and STEM‐HAADF (High Annular Angle Dark Field). The data acquired was treated using Digital micrograph software (Gatan) and FIJI software.

Regarding the µ‐chip battery (in situ), images were obtained at the end of the CV. After imaging, the µ‐battery cell and the µ‐fluid tubes were cleaned using DMC, and then methanol. The drying procedure was performed during the night in vacuum conditions.

For ex situ analysis, a Swagelok‐type battery (HC/Na) was assembled and studied in the same conditions as for in situ TEM imaging. After one cycle, the HC electrode was removed from the Swagelok cell in the dry room and rinsed several times with DMC. The images were acquired after the battery cycling process (post‐mortem) to compare those results to the in situ analysis.

### 2.5 GC/MS

The acquisition parameters in GC/MS (Thermo Scientific): For GC, a flow rate of 1.5 mL min^−1^. of He and a temperature gradient.^[^ ^15^ ^]^ For MS, an electronic impact (EI) source with an ionization energy of 70 eV. Use of a quadrupole (Q) to separate compounds based on their mass/charge ratio (m/z) (identification of compounds being processed with the National Institutes of Standards Library (NIST)).

## 3 Results and Discussion

### 3.1 Study of the Electrochemical System on the Anode Side (HC) – Half Coin Cell

**Figure 2:**
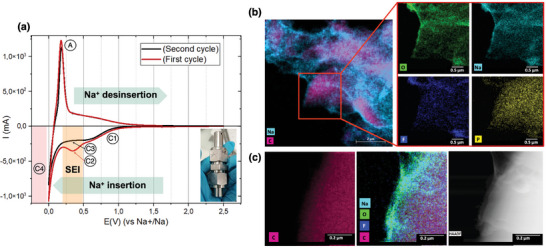
a) CV (voltage vs current) of a half coin cell at 1 mv s^−1^ with (A) anodic peak after extraction of Na^+^, (C1) Insertion of Na^+^ in HC, (C2) Formation of SEI, (C3) disappearing of the SEI formation cathodic pic during second discharge, and (C4) Na plating. b) EDX‐STEM of the hard‐carbon anode after the second cycle, showing Na plating. c) EDX‐STEM and HAADF of the SEI on the HC surface.

The analysis over half coin cell was performed to have a better understanding of the system before performing the analysis in the µ‐battery. As shown in **Figure** **2**a, CV allows us to observe different electrochemical reactions represented in three zones over two cycles. These correspond to the (dis)insertion of Na^+^ in the HC anode (green), the formation of the SEI (orange), and the Na plating on the anode surface (red). As the potential decreases from 2.5 V (Vs. Na^+^/Na) down to the anodic peak (A) at 0.2 V, the positive current increases significantly while Na^+^ is drawn from HC. Similarly, Na^+^ insertion takes place in the (C1) region while negative current increases toward high potential.

During the first discharge cycle, a cathodic peak (C2) was observed at 0.3 V, corresponding to the formation of the SEI. However, during the second discharge cycle, the cathodic peak (C2) disappeared as the reduction of the electrolyte (NP30) during the formation of the SEI in the first discharge cycle is so far irreversible.

In the work of L. Zhang et al.^[^ ^26^ ^]^ it was determined that the interaction between the electrolyte and metallic Na leads to the formation of gas in an open circuit. As a result, the coin cell battery presents two kinds of electrolyte degradation: Na/NP30 degradation and SEI formation, which explains the low cycling life in NIBs.^[^ ^27^, ^28^, ^29^ ^]^

As the potential gets closer to the standard potential of Na^+^/Na, the high current density on the anode surface and the low ionic conductivity of the HC for Na^+^ ions lead, as expected, to Na plating on the HC particle surface. This pronounced reduction in current corresponds to the (C4) peak in Figure 2a.

### 3.2 In Situ Analysis – µ‐Battery Electrochemical Behavior

**Figure 3:**
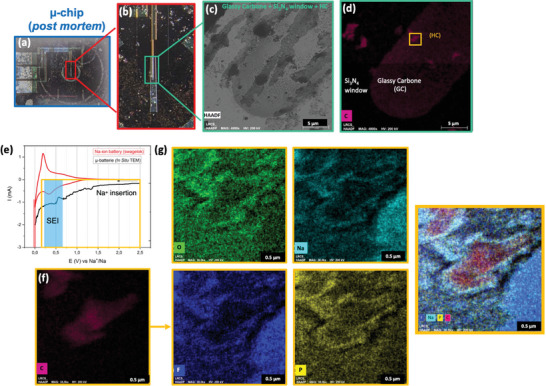
a) µ‐chip post‐mortem image obtained by optical microscope. b) Zoom in over WE. c) HAADF image over the WE. d) EDX‐STEM cartography over WE to reveal the HC particles. e) Voltammogram of coin cell and µ‐battery: Zone 1 (yellow): Continuous insertion of Na^+^ into the HC. Zone 2 (blue): Formation of SEI. Zone 3 (red): Plating deposit of Na. f) EDX‐STEM zoomed‐in image over an HC particle. g) EDX‐STEM image with detection of different elements (O, Na, F, P).

The µ‐battery was opened post‐mortem to characterize the HC anode after cycling in the optical microscope, as shown in **Figure** **3**a,b. The elements of the WE, such as the glassy carbon, Si_3_N_3_ window, and the earlier deposited HC anode, can be distinguished in the HAADF and EDX‐STEM, as presented in Figure 3c,d respectively. After cycling, the EDX‐STEM elemental cartography was performed on isolated HC particles on the glassy carbon WE to better understand the degradation/formation mechanisms. The analysis of SEI formation is presented in Figure 3f on one of the localized HC particles in Figure 3d region. The EDX‐STEM map reveals a layer surrounding the particle, composed of Na, O, F, and P, that presents a thickness of 389 nm (thickness measured using EDX‐STEM image analysis in FIJI software and gatan image scale bar as a reference). In addition, the CV curves of the coin cell and the µ‐battery follow similar trends, despite the instability of the current and the background noise. This background noise may have multiple sources, such as the electrolyte flow rate and the reference stability, as the electrochemical measurements were performed with a flow rate of NP30 of 1 µL min^−1^ and a sweeping rate of 1 mV s^−1^ with a Pt pseudo‐reference.

EDX‐STEM imaging reveals a higher concentration of Na around HC particles in comparison with the bulk of the HC particles as well. In the work of Z. Wei et al., it was found that SEI is mostly composed of organic and inorganic compounds, being organic compounds (sodium alkyl carbonates, RCH_2_OCO_2_Na) and inorganic products (Na_2_O, NaF), both Na‐rich compounds.^[^ ^30^ ^]^ Thus, the strong presence of Na around the HC anode particles may result from the SEI formation. Details about the possible chemical reactions resulting in the formation of several products of the SEI layer are presented in SI.^[^ ^21^, ^31^, ^32^, ^33^, ^34^, ^35^, ^36^ ^]^

Several hypotheses can be formulated: 1) Due to the lack of Na in the µ‐battery configuration, degradation of NP30 is not present at open circuit voltage, meaning that the electrolyte is degraded by SEI formation only during the cycling process. 2) Close to 0 V (vs Na^+/^Na), the sudden drop in current represents an interaction between e^−^ and Na^+^ for plating layer formation, which then reacts with the electrolyte to form a secondary SEI (SEI_bis_). The gas released by the interaction leads to the formation of a foam‐like structure, as shown further in Figure 5f.

Moreover, in Figure 3g, Na was found inside the HC particles. This observation points to the insertion of Na during cycling, in well‐agreement with the electrochemical curve. Na^+^ insertion in HC follows different mechanisms, such as sodium ion insertion on structural defects, Na^+^ ion intercalation between graphene layers, and micropore insertion, as presented in the work of H. Tonnoir et al.^[^ ^37^ ^]^

It is important to note that an electron beam can affect the SEI (Solid Electrolyte Interphase) by altering its morphology due to the degradation of organic species. During the EDX‐STEM acquisition, no changes were observed in the SEI layer over the HC particles. This qualitative observation suggests that the effects of the electron beam can be neglected. However, further experiments should be conducted in the same configuration to confirm this assumption.

### 3.3 Electrolyte Degradation Analysis

After studying the electrochemical systems of HC in the µ‐battery, the liquid product was recovered for analysis by GC/MS.

**Figure 4:**
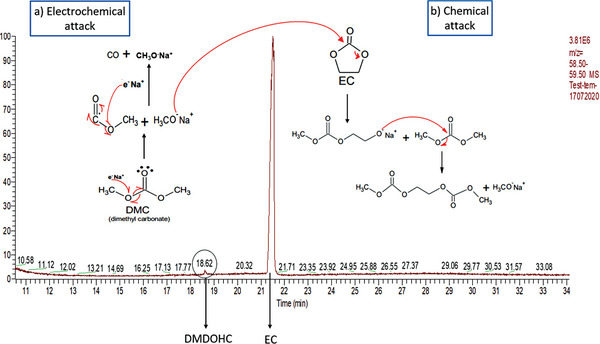
Chromatogram obtained for the liquid electrolyte after degradation. Specific mass m/z = 59, calibration for detection of DMDOHC. a) electrochemical attack carried by the e^−^ of Na atoms. b) chemical attack carried by the interaction of H_3_CONa (salt).

**Figure** **4** presents the chromatograph obtained, in which at 18.62 min DMDOHC (dimethyl‐2.5‐dioxahexane carboxylate plus sodium methoxide) is observed. The results are coherent with the literature.^[^ ^38^, ^39^, ^40^ ^]^ DMDOHC is a common degradation product in commercial batteries that use LP30 as an electrolyte: (LiPF_6_ at 1 mol/L in a mix of EC:DMC = 1:1 mass ratio).

The reaction mechanism between DMC/EC and Na is presented in Figure 4 as well. This is one of the possible paths that can lead to the formation of the degradation product. However, several different mechanisms can take place at the same time in parallel.^[^ ^15^ ^]^ The first step is an electrochemical reduction due to a one‐electron attack produced by Na e^−^ over an oxygen atom in DMC due to the electronegativity of O. This results in sodium methoxide formation (H_3_CONa) plus a free radical (C_2_O_2_H_3_). A second electrochemical attack takes place between the free radical and Na e^−^, giving as a result the liberation of CO plus sodium methoxide. A chemical reaction takes place as well between EC and sodium methoxide. The O in the methoxide attacks the C surrounded by 3 O, opening the molecule. The product of this chemical reaction reacts with DMC to obtain DMDOHC.^[^ ^41^ ^]^

As presented in the mechanism, DMDOHC is the result of the interaction between metallic Na and solvents in NP30, which means that during cycling, Na metal is formed and reacts immediately with the electrolyte. However, according to the electrochemical curve, metallic Na could be formed only close to 0 V (vs Na^+^/Na) (plating). A possible explanation is that during SEI formation, metallic Na exists without a plating process and reacts with solvents, meaning that SEI formation leads to DMDOHC formation, however, this hypothesis has to be proven by further analysis.

Due to the fact that Na can produce degradation, a replacement for coin cell, Swagelok, and the µ‐battery configuration is under study. NVP (Na_3_V_2_(PO^4^)_3_) is considered a suitable material for future analysis (especially because of its capacity to be used as anode or cathode material).^[^ ^42^, ^43^ ^]^ The most optimal for further studies and comparisons would be to have the closest materials configurations for both, coin cell and µ‐battery. For this reason, several deposition methods are under review by our group in order to make a proper deposition of NVP over platinum,^[^ ^44^, ^45^ ^]^ However, this step has to be addressed carefully due to the limitations imposed by the µ‐battery size.

Beam radiation was considered as a possible source of electrolyte degradation, this phenomenon has already been observed in other works.^[^ ^46^, ^47^ ^]^ To clarify this, the electrolyte was exposed to a direct beam in STEM mode with a total radiation dose of 1 × 10^8^ e^−^/Å^2^, and the resulting product was studied via GC/MS. The resulting chromatogram is presented in Figure S3 (Supporting Information). The presence of CH_3_CN, an impurity suspected to be present in the chromatography column, was noted. EC was also found, however, no other products were detected after beam radiation. This result allows us to conclude that the interaction of the electron beam can be negligible during in situ cycling.

### 3.4 Na plating

**Figure 5:**
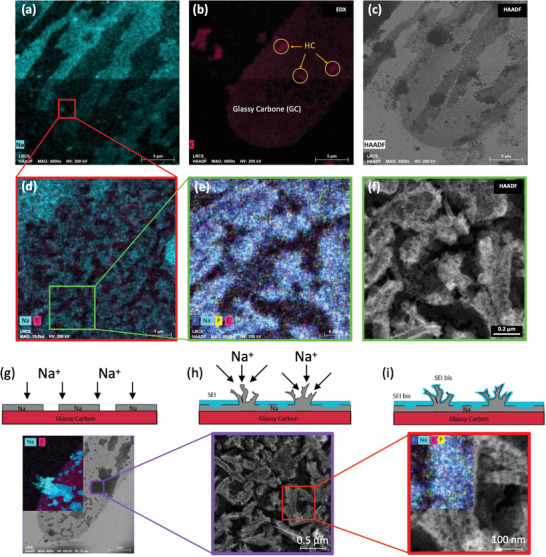
EDX‐STEM maps of a) Na and b) Carbon, and c) HAADF analysis over the same area of the WE. d) Zoom in a Na‐poor zone which reveals a dendritic kind of structure. e) EDX‐STEM analysis at higher magnification of image d where a dendritic kind of structure is observed. f) HAADF over the same zone as image d where foam‐type structure is founded. Schematic representation of electrochemical reactions occurring on the surface of glassy carbon electrode with STEM images associated reactions g) non‐uniform Na plating, h) dendrite and SEI formations, and i) SEI_bis_ formation.

As shown in **Figure** **5**, an uneven distribution of Na is observed over the WE. The EDX‐STEM analysis over the edge of the electrode reveals a dendritic‐like structure with a low concentration of Na, as shown in Figure 5d. The morphology can be better appreciated in the magnified HAADF image, revealing the formation of a foam‐like structure in the dendrites, as shown in Figure 5f. In addition, the EDX‐STEM analysis of the structure shows the presence of the same elements as the SEI layer.

During Na plating, a non‐uniform layer of metallic Na is deposed over the glassy carbon WE, as shown in Figure 5g. The formation of this layer creates different nucleation points, which leads to the formation of dendrites shown in Figure 5h. Then, the metal reacts with the electrolyte and forms the SEI_bis_ mentioned previously, as shown in Figure 5i. The presence of metallic Na as well as the formation of the SEI_bis_ liberates gases that contribute to the formation of the foam‐like structure. The SEI_bis_ is formed over the dendrites that are the substrate for the protective layer and adopts its form. Even though the SEI_bis_ is conductive for ions, the foam structure reduces its conductivity and as a consequence the performance of the battery. In this case, during plating, the two kinds of electrolyte degradation processes of the half‐coin cell are present.

### 3.5 Beam Influence Over the SEI Obtained During Cycling Was

#### 3.5.1 Ex Situ Analysis – Swagelok Electrochemical Behavior

As shown in Figure S1a (Supporting Information), electrochemical analysis was carried out on a Swagelok‐type battery after one cycle (post‐mortem) to compare the results with the in situ analysis. The cycling was performed with the same parameters as for the µ‐chip. The voltammogram presented in Figure S1b (Supporting Information) shows similar trends for Swagelok, coin cell, and µ‐battery curves. These similarities in the electrochemical behavior allow us to compare the ex situ and in situ results for validation. In addition, the counter electrode for Swagelok was metallic Na, meaning that the two kinds of electrolyte degradation present in the half coin cell configuration may be present as well.

As shown in Figure S2 and Table S1 (Supporting Information), two major species, Na and C, are detected over an HC particle, according to the EDX‐STEM analysis. Furthermore, the detection of Na inside the C particle confirms the insertion of Na in HC. A zoom‐in over the HC particles reveals a layer formed of various species, as shown in Figure S2f (Supporting Information). This result is coherent with the EDX‐STEM of the in situ analysis, confirming the formation of an SEI layer surrounding the HC anode particle, as the layer is composed of the same elements found in the SEI for the in situ analysis. However, the thickness of the SEI is only 83 nm (thickness measured using EDX‐STEM image analysis in FIJI software and Gatan GMS image scale bar as reference) in the Swagelok battery, a considerable reduction in comparison with the µ‐battery case. The lower thickness of the SEI in the Swagelok configuration can be explained by the difference in current distribution during cycling. Indeed, the necessary pressure applied in the Swagelok to enhance the contacts between the different battery components induces a significant variation in the current distribution in comparison with the µ‐battery configuration. A zoom‐in on the EDX‐STEM cartography of the HC particle shows the chemical contrast between the HC and the SEI, as shown in Figure S2f (Supporting Information).

In the literature, it is reported that the SEI layer in NIB is less thick than the SEI found in LIB.^[^ ^48^, ^49^ ^]^ Indeed, Na salts are more soluble in organic solvents than Li salts. In NIBs, it takes a larger number of cycles or higher potential to obtain a stable SEI layer. In the work of M. Á. Muñoz‐Márquez et al.,^[^ ^50^ ^]^ it is presented that the SEI layer in NIB is not thicker than 7 nm. In contrast, our work showed a thicker SEI layer. An explanation for this phenomenon is the fact that the SEI layer formed during cycling is porous due to gas formation, which increases considerably its size with the liquid electrolyte swelling.

## 4 Conclusion

NIBs exhibit great promise for future applications in the field of energy storage due to the abundance and low cost of their base materials, as well as their environmentally friendly properties. However, in order to progress toward commercialization, it is crucial to go deeper into the limitations understanding of NIB.

In this study, we employed the coupling of liquid (ec‐TEM) and GC/MS to gain a comprehensive understanding of SEI formation and the degradation of liquid electrolyte NP30. This combined approach served as a unidirectional analysis method, enabling us to track the battery's cycling behavior and enhance our comprehension of the chemical and electrochemical phenomena involved.

To summarize, we used a liquid electrochemical cell for in situ µ‐battery measurements, a coin cell battery with an HC anode as an electrochemical reference, and a Swagelok battery for ex situ measurements to validate the in situ analysis. Electrochemical analysis revealed three distinct stages of behavior during the initial cycle: Na^+^ insertion, SEI formation, and Na plating near 0 V. The CV data demonstrated the coherence of the in situ and ex situ experiments with the coin cell reference, showcasing similar electrochemical behaviors. Additionally, in situ EDX‐STEM maps confirmed the formation of the SEI layer around HC particles, aligning with the electrochemical curves. Moreover, GC/MS analysis detected the presence of DMDOHC, a product resulting from NP30 electrolyte degradation, which was consistent with previous findings in the literature. Our findings indicated that the formation mechanism of DMDOHC from electrolyte degradation leads to the generation of gases such as CO, contributing to the foam‐like morphology of Na plating as observed through EDX‐STEM and confirmed by ex situ results. Furthermore, the formation of SEI through this process further reduced the battery's performance.

The successful coupling of ec‐TEM and GC/MS proved advantageous, as it minimized disturbances in imaging and electrolyte analysis. This analytical synergy holds great potential for future research endeavors, offering an improved understanding of SEI formation, electrolyte degradation, and the formulation of new NIB configurations to enhance battery performance.

Further investigations are warranted to deepen our understanding of SEI formation and electrolyte degradation in NIB. Specifically, the analysis of gases should be conducted to confirm the presence of the predicted degradation products, and in situ analysis at different states of charge (SoC) should be performed to gain insights into the dynamics of SEI formation. Moreover, employing the same methodology and configuration with different electrolytes will facilitate the optimization of the anode/electrolyte pairing for NIB.

## Conflict of Interest

The authors declare no conflict of interest.

## Author Contributions

A.D. conceived and supervised the research. A.D., G.G., and F.R. designed the experiments. M.T. and N.F. performed most of the in situ TEM experiments, including characterizations and measurements. K.G.M., N.F., H.T., D.H., G.G., and A.D. analyzed and interpreted the TEM and GC/MS data. The paper was written by K.G.M., N.F., and A.D. with contributions from all of the coauthors.

## Supporting information

Supporting Information

## Data Availability

The data that support the findings of this study are available from the corresponding author upon reasonable request.
