# Supplementary material for: Coupling Liquid Electrochemical TEM and Mass‐Spectrometry to Investigate Electrochemical Reactions Occurring in a Na‐Ion Battery Anode
Source: Small Methods. 2024 Aug 29;8(12):2400365. doi: 10.1002/smtd.202400365 (PMC11672185; doi:10.1002/smtd.202400365)
Supplement: Supplementary file 1 — Supporting Information [file SMTD-8-2400365-s001.docx]

**Supporting information:**

**Coupling liquid electrochemical TEM and mass-spectrometry to investigate electrochemical reactions
occurring in a Na-ion battery anode**

Kevyn Gallegos Moncayo^1,2^, Nicolas Folastre^1,2^, Milan Toledo^1^, Hélène Tonnoir^1,2^,
François Rabuel^1,2^, Grégory Gachot^1,2^, Da Huo^1,2^, Arnaud Demortière^1,2,3*^

^1^Laboratoire de Réactivité et Chimie des Solides (LRCS),
CNRS UMR 7314, UPJV, Hub de l’Energie, 15 rue Baudelocque, 80039 Amiens Cedex, France.
^2^Réseau sur le Stockage Electrochimique de l’Energie (RS2E),
CNRS FR 3459, Hub de l’Energie, 15 Rue Baudelocque, 80039 Amiens Cedex, France.
 ^3^ALISTORE-European Research Institute,
CNRS FR 3104, Hub de l’Energie, Rue Baudelocque, 80039 Amiens Cedex, France.

*Corresponding author: **arnaud.demortiere@cnrs.fr**


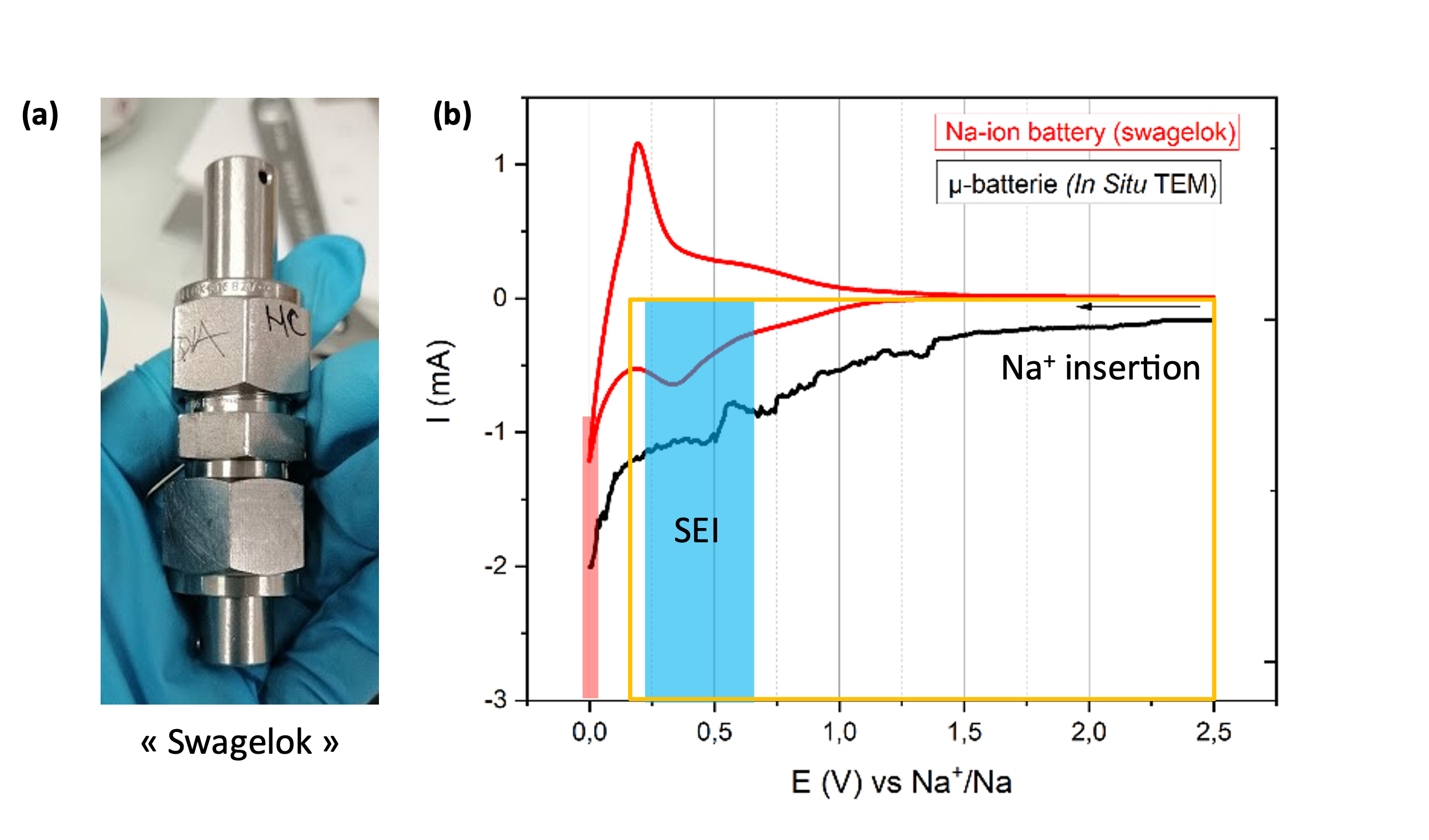


**Figure S1:** (a) Swagelok cell configuration for cycling and post-mortem analysis, (b) Electrochemical curve for the Swagelok and the µ-battery experiments (voltage vs current). The shape of the discharge curve is similar for both configurations.


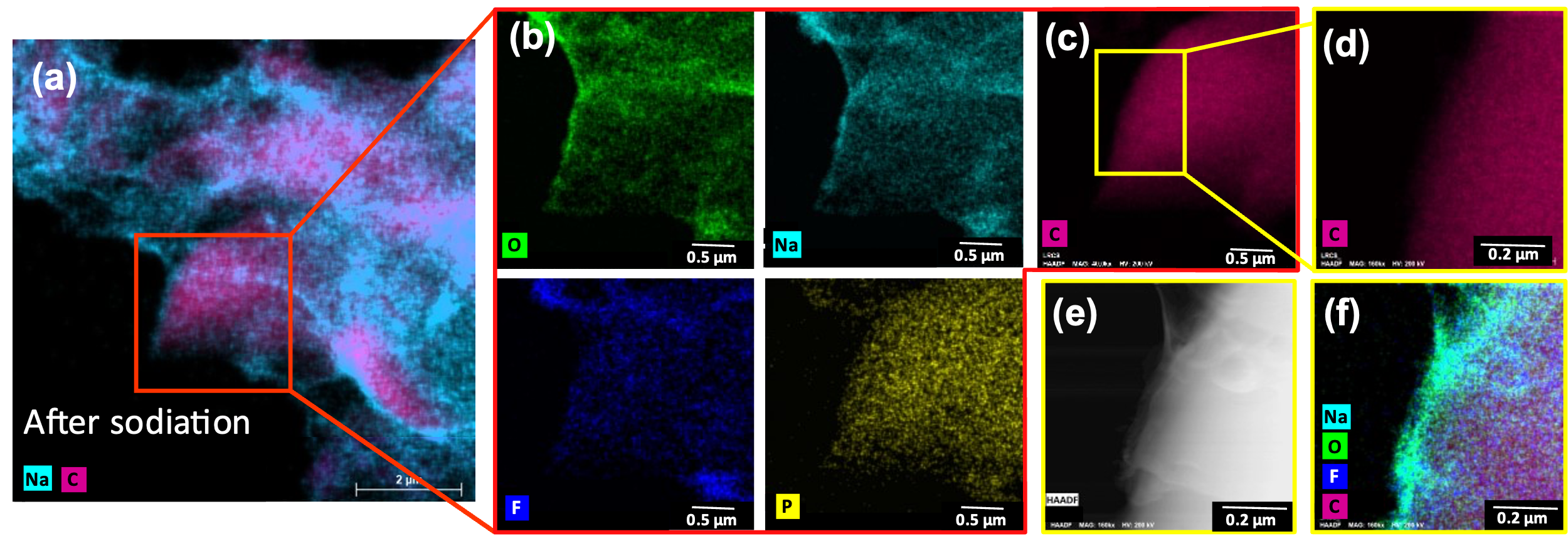


**Figure S2:** *(a) TEM-EDX analysis over a particle obtained after cycling in Swagelok. (b) EDX zoomed-in image over an HC particle to represent the different species present in the particle and its surroundings, (c) HC particle (EDX view) to highlight the Carbon domain, (d) EDX image over the edge of HC particle, (e) HAADF TEM image of the SEI layer and HC edge for the determination of SEI thickness, (f) EDX image of the SEI layer surrounding the HC particle.*

**Table S1:** Semi-quantitative values for TEM-EDX analysis over SEI post-mortem Swagelok battery configuration. The SEI represents 11.8 % of the total imaged surface of the HC particle.

| Element | Mass ratio | Atomic ratio |
| --- | --- | --- |
| Oxygen | 33.36 % | 49.93 % |
| Sodium | 43.16 % | 35.04 % |
| Phosphate | 04.61 % | 08.18 % |
| Fluorine | 10.33 % | 06.84 % |

Detail of reduction reactions for the SEI formation.

EC reactions

EC+Na^+^ +e^-^ 🡪 C_2_H_4_OCO_2_^-^ + Na^+^ (1)

EC +2Na^+^ + 2e^-^ 🡪 Na_2_CO_3_ + C_2_H_4_ (2)

EC+2e^-^ 🡪 OC_2_H_4_O_2_^-^ + CO (3)

DMC reactions

DMC +e^-^ +Na^+^ 🡪 CH_3_. + CH_3_OCO_2_Li (4)

DMC +e^-^ +Na^+^ 🡪 CH_3_ONa. + CH_3_OCO. (5)

H_2_O traces reaction

H_2_O + e^-^ + Na^+^ 🡪 NaOH + 1/2H_2_ (6)

LiOH + e^-^ +Na^+^ 🡪Na_2_O + 1/2H_2_ (7)

H_2_O + (C_2_H_4_OCO_2_Na)_2_ 🡪Li_2_CO_3_ + CO_2_ + (CH_2_OH)_2_ (8)

CO_2_ reaction

2CO_2_ +2e^-^ +2Na^+^ 🡪Li_2_CO_3_ + CO (9)

NaPF_6_ reactions

NaPF_6_ ↔ NaF +PF_5_^-^ (10)

PF_5_^-^ + H_2_O 🡪 2HF +PF_3_O (11)

PF_5_^-^ +ne^-^ + nNa^+^ 🡪NaF +Na_x_PF_y_ (12)

PF_3_O +ne^-^ + nNa^+^ 🡪NaF + Na_x_POF_y_ (13)


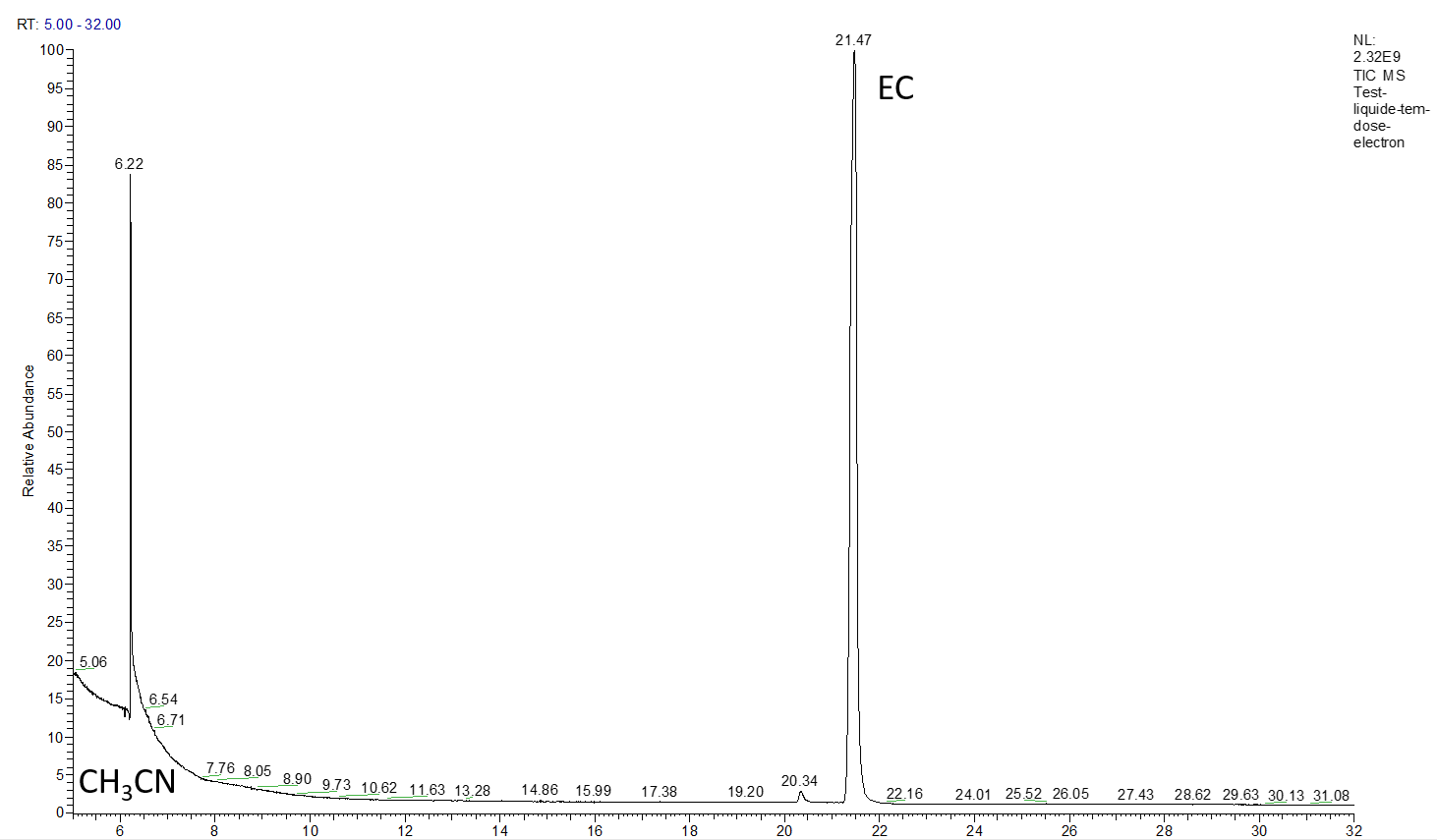


**Figure S3:** Chromatogram obtained for the liquid electrolyte after beam exposure for the study of the effects of electron beam radiation over NP30 electrolyte.
